# Multivisceral resection of pancreatic neuroendocrine tumours: a report of two cases

**DOI:** 10.1186/1477-7819-9-93

**Published:** 2011-08-22

**Authors:** Justin S Gundara, Raul Alvarado-Bachmann, Nicholas Williams, Sivakumar Gananadha, Anthony Gill, Thomas J Hugh, Jaswinder S Samra

**Affiliations:** 1 Department of Gastrointestinal Surgery, Royal North Shore Hospital, University of Sydney, St Leonards NSW 2065, Australia; 2 Department of Anatomical Pathology, Royal North Shore Hospital, University of Sydney, St Leonards NSW 2065, Australia

**Keywords:** pancreatoduodenectomy, neuroendocrine, pNET, islet cell carcinoma, GEP-NET

## Abstract

Pancreatic neuroendocrine tumours (pNETs) are rare and surgical resection offers the only possibility of cure for localised disease. The role of surgery in the setting of locally advanced and metastatic disease is more controversial. Emerging data suggests that synchronous surgical resection of pancreas and liver may be associated with increased survival. We report two cases of synchronous, one stage multivisceral resections for pNET and associated reconstruction. We highlight the technical issues involved in such extensive resections and demonstrate that one stage multivisceral operations can be achieved safely.

## Background

Pancreatic neuroendocrine tumours (pNETs) are relatively rare with an annual incidence of two to three cases per million of population [1]. Such tumours can be classified as functional or non-functional. In earlier studies, functional tumours were more common than non-functional tumours. However, more recent data suggests that up to 85% of pNETs are non-functioning [1,2]. Patients with functional tumours usually present earlier due to unique clinical symptoms caused by hormone hypersecretion. In contrast, non-functioning tumours present later with non-specific symptoms and patients often have metastatic disease at the time of diagnosis [3].

TNM staging, the modified WHO classification and Ki-67 proliferative index may predict recurrence, but are less useful in individual cases due to the unpredictable nature of this disease [4]. For a solitary pNET, resection remains the best option for long term cure [3]. Retrospective studies also suggest that synchronous resection of the primary and metastatic liver disease is also associated with improved survival outcomes [3,5,6].

Surgical options in the presence of locally advanced disease are more controversial however. Current clinical guidelines recommend aggressive surgical treatment [7]. However, these patients typically require complex, technically demanding resections that push the boundaries of not only technical feasibility, but also acceptable morbidity and mortality.

Whilst there is mounting evidence justifying such a radical approach [3,5,6,8], prospective, multi-centre studies reporting disease free and functional quality of life survival outcomes do not presently exist for this sub-group of pNET patients, thus making clinical decision making problematic. We highlight two further examples of large pancreatic neuroendocrine tumours requiring multivisceral resection to demonstrate that complex one stage operations can be achieved safely.

## Case Report 1

A 63 year old woman presented with a six month history of progressive upper abdominal discomfort and intermittent vomiting. History was significant only for left breast cancer for which she had undergone a mastectomy seven years earlier.

Clinically she possessed a firm right upper quadrant, tender mass. Blood tests showed mildly deranged liver function tests (ALP: 279IU/l; GGT: 282IU/l) and an elevated serum chromogranin A level (CgA; 52IU/l; range: 0-17.2). Computed tomography (CT) of the abdomen showed a well demarcated head of pancreas lesion (4 × 4 cm) and a large heterogeneous right hemi-liver lesion (15 × 15 × 12 cm). Both lesions showed uptake on a subsequent octreotide scan. An endoscopic ultrasound was also performed and fine needle biopsies of both pancreas and liver lesions were shown to be consistent with a diagnosis of neuroendocrine tumour. Laparoscopy was negative for further dissemination of disease and the multi-disciplinary oncology team meeting consensus was in favour of surgical resection.

**Figure 1:**
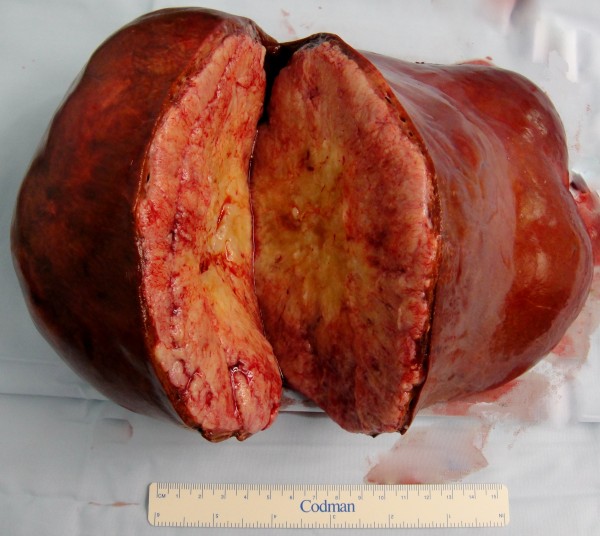
**Macroscopic view of extended right hepatectomy specimen (Case 1)**.

A midline laparotomy was performed and an extended right hepatectomy commenced. Following mobilisation of the right colon, the duodenum was Kocherised. The avascular plane anterior to the inferior vena cava (IVC) was dissected and a nylon tape passed behind the liver. This facilitated ultrasonic dissector division of hepatic parenchyma with the "hanging manoeuvre". Inflow occlusion was not necessary. At this point, the right and middle hepatic veins were divided. The right portal vein was transected and oversewn transversely to avoid main portal vein trunk stenosis. This completed an extended right hepatectomy (segments 1, 4a, 5, 6, 7 and 8) and the specimen was removed (Figure 1).

**Figure 2:**
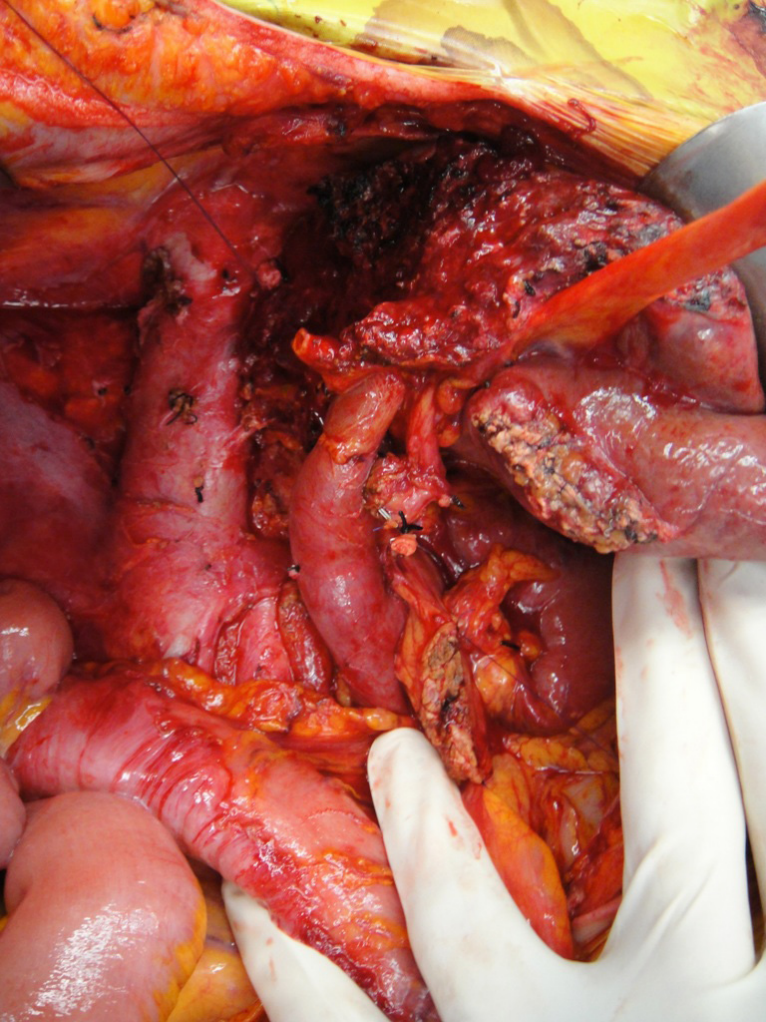
**Operative resection bed following removal of right liver and head of pancreas tumours (Case 1)**.

This was followed by a pancreatoduodenectomy. The lesser sac was entered, the infra-colic compartment examined and tumour mobility (within context of portal vein and superior mesenteric vasculature) assessed. Following confirmation of resectability, the stomach antrum and common hepatic duct were divided sequentially. The neck of pancreas was then transected. The proximal jejunum was divided and the ligament of Treitz dissected to mobilise and remove the specimen from the abdomen. Figure 2 shows the resection bed following removal of liver and pancreas tumours. Reconstruction involved a double layered, end-side pancreatico-jejunostomy, an end-side hepatico-jejunostomy and a side-side gastro-jejunostomy.

The abdomen was drained and closed. Total operative time was 6.5 hours with an estimated blood loss of 750 ml. The post-operative period was complicated by an intra-abdominal collection which was managed with percutaneous drainage.

Histopathological examination showed a well differentiated pancreatic neuroendocrine carcinoma 45 mm in diameter, with a mitotic rate of nine mitoses per 10 high power fields (hpf) and a Ki-67 proliferative index of 15%. All microscopic margins were clear. A completely excised single liver metastasis, 158 mm in diameter, was identified in the hepatectomy specimen with associated cytological atypia and focal coagulative necrosis. None of 33 resected lymph nodes were involved.

The patient remains well two years following resection. Serial CT scans (at 3, 6, 12 and 24 months) showed no evidence of recurrence and CgA levels are normal.

## Case Report 2

A 60 year old woman presented with a twelve month history of fatigue, anorexia, weight loss and abdominal distension. She had a history of well controlled hypertension and type II diabetes mellitus.

**Figure 3:**
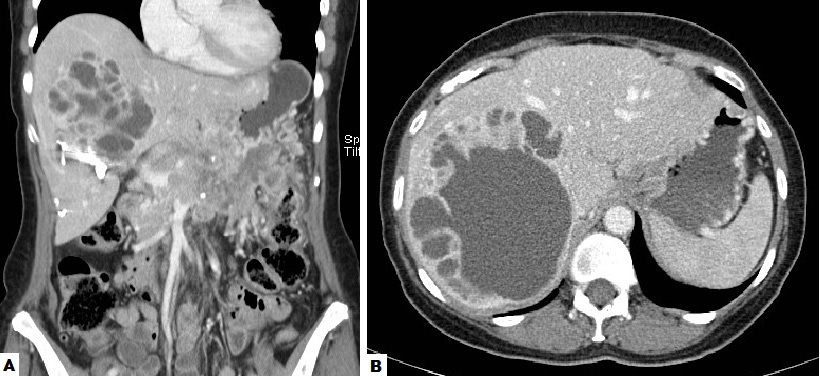
**a: CT demonstrating pancreatic mass with superior mesenteric/portal vein encasement and associated liver metastasis (Case 2); b: CT demonstrating right liver metastasis (post embolization; Case 2)**.

Liver function tests were slightly abnormal (ALP: 383IU/l; GGT: 216IU/l). CT of the abdomen demonstrated a large pancreatic mass (13 × 9 × 5 cm) compressing the confluence of the portal and superior mesenteric veins (Figure 3a). The right colon and antrum of the stomach also appeared to be intimately involved with the tumour. Additionally, a 12 cm diameter mixed cystic/solid mass was noted to occupy the majority of the right hemi-liver (Figure 3b). Her serum CgA level was elevated at 507IU/l (range: 0-17.2) and an octreotide scan showed avid uptake within the pancreatic mass and within the periphery of the liver lesion.

Laparoscopy was performed to exclude additional peritoneal disease and biopsies of the right liver tumour were taken. Biopsy specimens confirmed the diagnosis of a neuroendocrine tumour with a Ki-67 index of 4%.

At a multidisciplinary oncology team meeting, consensus of opinion was that the patient should be offered resection. Volumetric analysis demonstrated a 24% future remnant liver volume. A right portal vein embolisation was performed with a view to inducing left lobe hypertrophy. Four weeks later, reassessment of the liver volume confirmed that the future left lateral section remnant volume had increased to 32%.

A midline laparotomy was performed. Exploration confirmed that the pancreatic mass had invaded into the greater curvature of the stomach and adjacent transverse colon. Initially, an extended right hepatectomy (segments 4a, 5, 6, 7 and 8) was performed including excision of the terminal part of the middle hepatic vein flush with the IVC. The left hepatic duct was divided, and the right hepatic artery was divided 1 cm distal to its confluence with the left hepatic artery. An extended Kocher's manoeuvre was performed and the posterior relations of the mass were assessed. It was evident that while the IVC and aorta were free from disease, the portal vein (PV) and coeliac axis were involved by tumour and would require resection, en-bloc with the mass. The superior mesenteric vein (SMV) and artery (SMA) were identified in the infra-colic compartment and the dissection plane was maintained along the SMA to its aortic origin. The right colon and small bowel were mobilised using the Cattell-Braasch manoeuvre [9].

**Figure 4:**
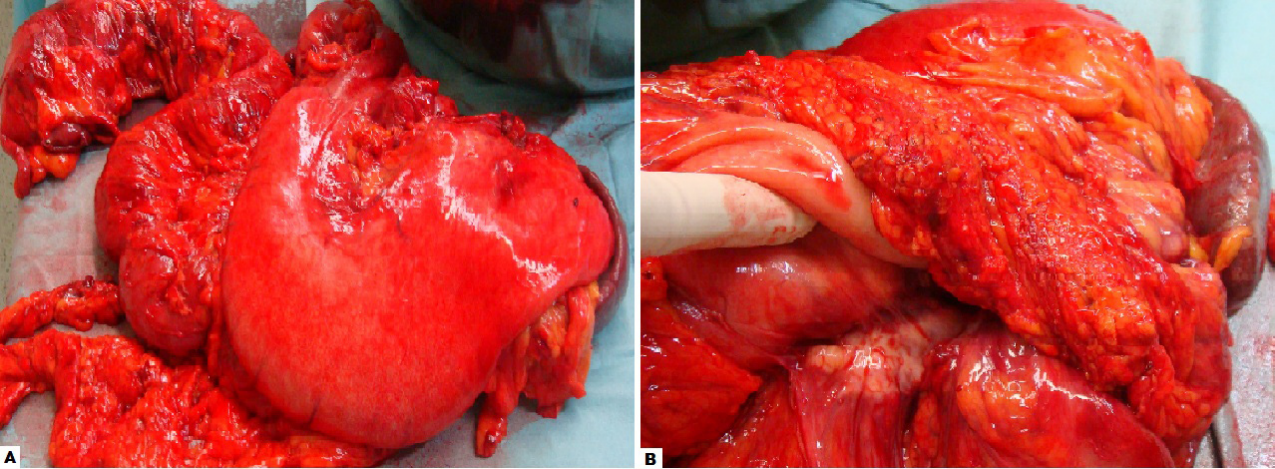
**a: Macroscopic view of en bloc primary tumour resection specimen (Case 2); b: pancreatic tumour invasion of lesser sac and posterior stomach wall (Case 2)**.

**Figure 5:**
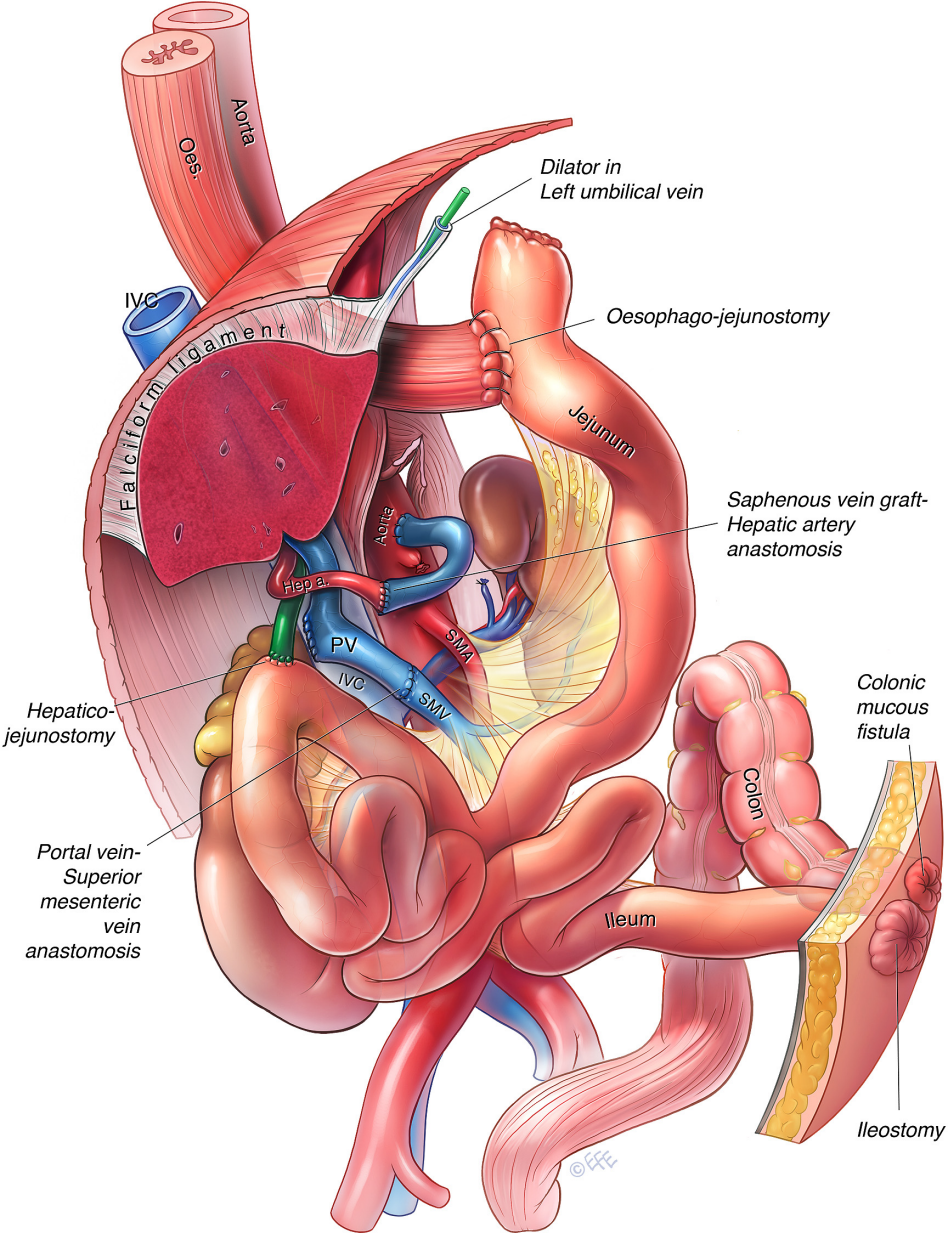
**Illustration depicting vascular and enteric reconstruction post resection (Case 2)**.

The involved PV and SMV were then transected above and below the mass, respectively. Continuity was restored by direct end to end anastomosis; facilitated by the extra mobility gained from the preceding hepatic resection and small bowel mesenteric mobilisation. Following this, an interposition saphenous vein graft was placed from the aorta to the junction of the right and left hepatic artery. The common hepatic artery was divided and the coeliac axis was divided and ligated flush with the aorta. The dissection plane was now continued to the left of the aorta along Gerota's fascia. The left adrenal gland was adherent to the tumour and was included in the en-bloc specimen. The terminal ileum, descending colon and gastro-oesophageal junction were all divided, thus completing the resection which consisted of the stomach, spleen, pancreas, duodenum, left adrenal, right colon and transverse colon (Figure 4a, b). Reconstruction consisted of a oesophago-jejunostomy and hepatico-jejunostomy (Figure 5). Finally an end ileostomy and colonic mucous fistula were fashioned on the left abdominal wall. The total operative time was 16 hours and the intraoperative blood loss was 1850 mls.

**Figure 6:**
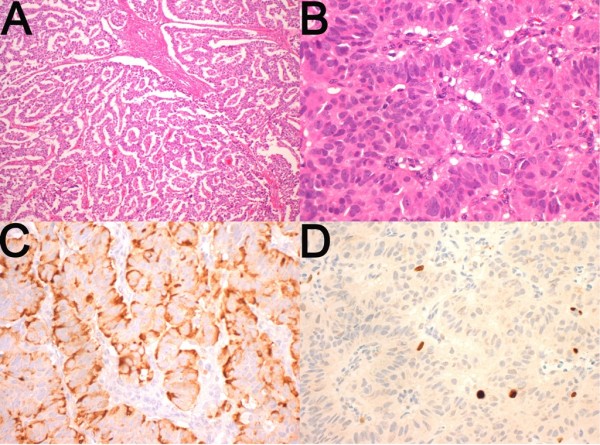
**a: at low magnification the pancreatic tumour displayed a typical trabecular architecture; b: at higher power the typical neuroendocrine nuclear features characterised by dispersed chromatin are observed; c: Immunohistochemistry for Chromogranin was diffusely strongly positive; d: Immunohistochemistry for Ki-67 demonstrated a proliferative index of 2% (original magnifications a: 100×, b, c, d: 400×; Case 2)**.

Histopathological examination revealed a well differentiated pancreatic neuroendocrine carcinoma 95 mm in diameter with a mitotic rate of one mitosis per 10 hpf and a Ki-67 proliferative index of 2% (Figure 6). The tumour demonstrated local invasion into the retroperitoneum, colon, stomach and left adrenal gland, but all microscopic margins were clear. A completely excised single liver metastasis, 90 mm in diameter, was found in the hepatectomy specimen, and two out of 24 lymph nodes were involved by metastatic carcinoma.

The post-operative course was complicated by refractory chylous ascites, which was successfully managed with a peritoneo-venous shunt on the twenty fourth post-operative day. She was discharged from hospital without any further complications.

Follow up showed a good functional recovery from surgery with independent resumption of activities of daily living by one month. CT at three and six months showed post-operative changes only. Nine months after surgery, the patient began to complain of left subscapular chest wall pain. A gallium 68 scan confirmed recurrence of tumour in the ribs bilaterally, mediastinum and in the remnant left liver. Slow release octreotide therapy was commenced and transarterial chemoembolisation (TACE) therapy was pursued for local control of hepatic disease. Bony disease was treated with radiotherapy.

Disease appeared static until 12 months. Systemic chemotherapy was commenced upon medical oncology advice with everolimus. Unfortunately, she developed severe haematological and renal complications as a consequence and died 15 months after her initial operation.

## Discussion

Successful multivisceral resections of this magnitude have not been previously described. The low incidence, variable biological behaviour of pNETs and a reluctance to undertake multi-visceral resections for advanced disease have been significant impediments to publishing large volume, prospective therapeutic studies.

Despite this, the current European Neuroendocrine Tumour Society (ENETS) guidelines support "aggressive surgery" where tumours larger than 2 cm and/or locally advanced disease may necessitate en-bloc resection of adjacent organs [7]. Whilst such guidelines are based upon relatively small retrospective studies [5,6], they demonstrate that a successful outcome is possible following resection of limited locally advanced disease with acceptable morbidity and mortality. Hellman et al. (2003) conclude that "conventional contraindications to surgical resection, such as superior mesenteric vein invasion and nodal or distant metastases, should be reconsidered in patients with advanced neuroendocrine tumors".

Given potential morbidity, mortality and the lasting impact that such operations may have upon patients, a survival advantage needs to be demonstrated to justify aggressive management. Retrospective analyses have shown some survival benefit following surgery for locally advanced disease [6,10,11]. However, as articulated in the ENETS guidelines, this evidence suffers from a heterogeneous patient/tumour cohort (various stages of disease; mixed functioning/non-functioning tumours) and multi-modality treatment strategies that make conclusions regarding aggressive surgery specifically, difficult to deduce.

With regard to liver metastases, a number of studies have shown that combined resection of the primary lesion and small volume metastatic liver disease improves survival outcomes [6,8,12]. ENETS guidelines site the possibility of recurrent liver disease and suggest that resection should only be pursued if at least 90% of the tumour volume can be removed [7]. Resecting the primary while leaving hepatic metastases in situ does not confer a survival advantage and should not be undertaken [13].

Management of inoperable metastatic liver disease may involve a spectrum of multi-modality therapy. In highly selected cases even liver transplantation may be considered [14], but most patients generally possess disease only amenable to loco-regional ablative therapies (such as TACE) or systemic treatment. The use of somatostatin analogues have traditionally been employed and significantly slow disease progression in non-functioning disseminated pNETs [15]. More recently, the tyrosine kinase inhibitor sunitinib has demonstrated Phase 3 trial efficacy in management of disseminated pNET, leading to prolonged progression and treatment free survival [16].

Both cases demonstrate that a complex multivisceral resection with synchronous hepatectomy can be performed safely, provided that the surgeon executes the multistep procedure in an appropriate sequence, in order to avoid the many potential pitfalls. For example, a staged approach with initial extended hepatectomy may have made a subsequent laparotomy and dissection in the supracolic compartment more hazardous. More importantly, in Case 2, initial resection of the primary lesion may have rendered subsequent attempts at inducing future remnant liver hypertrophy ineffective, due to the absence of the trophic effect of endogenous insulin. Early liver resection also aided further dissection as it provided increased manoeuvrability and facilitated selective control of the portal vein without further need for a Pringle manoeuvre. Additionally, sequential resection/reconstruction of the portal vein and coeliac axis minimised hepatic ischaemia. Had prolonged portal vein and coeliac axis clamping been required, the obliterated umbilical vein could have been used as a potential bypass conduit.

Chylous ascites was a predictable complication in Case 2, given such extensive retroperitoneal dissection. In this situation we favoured a peritoneo-venous shunt over repeated peritoneal taps to lower the risk of infection.

The natural history of pNETs continues to be difficult to predict despite advances in staging, grading and classification systems. A lack of consensus within methods of pathology reporting has also been highlighted recently [17] and serves only to make prognostication even more complex. To enhance clinical decision making utility, these systems have recently been rationalised by ENETS in the form of clinical guidelines for investigation and management [7].

Whilst further histopathological and prognostic criteria such as the Ki-67 proliferative index and mitotic count are included, such markers may still underestimate the unpredictable nature of this disease [4]. Contrasting proliferative markers of the two presented cases demonstrates this point. Both cases possessed well differentiated primary tumours. Case 1 however, showed a much higher mitotic rate (9 vs 1 mitoses per10 hpf) and Ki-67 index (15 vs 2%). Thus, despite histological evidence of relatively indolent tumour biology, Case 2 ultimately possessed a more aggressive tumour clinically, leading to early recurrence despite a margin negative resection. Although proliferative markers have been validated and correlate with prognosis [18], our current understanding of pNET tumour biology at a molecular level demands further attention to explain tumour heterogeneity. This will be necessary before translational benefits (such as validated biomarkers to assist diagnosis, treatment and prognostication) can be derived.

Clinical decision making therefore remains difficult in individual cases and deciding which patients should be offered resection continues to challenge experienced clinicians [7]. Beyond tumour biology and technically achievable surgical resection, the clinician must also bear in mind patient co-morbidity, post-operative quality of life and preference when considering management options.

## Conclusion

Complex multivisceral resections of neuroendocrine tumours can be achieved safely with appropriate preoperative planning and surgical expertise. We advocate resection of primary and secondary liver disease in a one stage procedure where patient co-morbidity and technical expertise allow. Further studies are required to justify and standardise the approach to aggressive surgery for locally advanced disease.

## Consent

Written informed consent was obtained from patients for publication of Case reports and any accompanying images. A copy of the written consent is available for review by the Editor-in-Chief of this journal.

## Competing interests

The authors declare that they have no competing interests.

## Authors' contributions

JS, TH, RA and JG were involved in the clinical care of patients. JG, JS and NW collected clinical data. AG reported pathological findings and prepared slides for manuscript inclusion. JG, RA, NW, TH, SG and JS drafted the manuscript. All authors were involved in editing and final review.
